# Supplementary material for: Are carbon emissions trading and green financial instruments synergistic? -Comprehensive quantitative research based on content analysis
Source: PLoS One. 2024 Mar 7;19(3):e0298601. doi: 10.1371/journal.pone.0298601 (PMC10919601; doi:10.1371/journal.pone.0298601)
Supplement: S1 Data — (ZIP) [file pone.0298601.s001.zip › Data/地级市Z_F工作报告.zip!/║┌┴·╜¡/╝a╬≈╩╨/└┤╫╘╣1⁄2╓┌║┼í╛┬φ┐╦╩2╛▌═°í┐_2018.docx]

各位代表：

现在，我代表市人民政府，向大会报告工作，请予审议。

一、2017年工作回顾

2017年，是鸡西转型发展取得新进展、民生事业取得新进步的一年。一年来，在省委、省政府和市委的正确领导下，我们全面贯彻落实党的十九大精神和习近平总书记系列重要讲话精神，聚焦转型发展兴市富民，攻坚克难，砥砺奋进，圆满完成市人大十五届一次会议确定的目标任务。

经济持续稳中向好。预计，全市地区生产总值同比增长6.5%；规模以上工业增加值增长8%；固定资产投资增长8%；社会消费品零售总额增长8.5%；一般公共预算收入增长6%；外贸进出口总额增长20%；城乡居民人均可支配收入均增长6%。

发展动能集聚释放。启动实施5000万元以上重点产业项目89个，其中投资22.4亿元的唯大（鸡西）石墨烯产业园、10.5亿元的贝特瑞石墨产业园、5亿元的珍爱紫苏油等一批项目快速推进，梨树风电场、鸡东故香米业、密山久鼎水上乐园等27个项目如期竣工。坚持向引进战略投资者要发展，引入首旅集团、中科遥感集团等中国500强和行业领军企业，全年签约项目101个，当年开工建设71个，实际到位资金60.7亿元。坚持向高新技术成果产业化要发展，组建了全省北药产业技术创新战略联盟，建设科技企业孵化器和众创空间30个。新注册成立科技型企业164家，新增主营业务收入500万元以上科技型企业25家，同比分别增长59.2%、78.6%。高新技术产业产值实现73.1亿元，同比增长9.1%。珍宝岛药业、奥宇石墨在首届省政府质量奖评选中获提名奖。对上争取再创新高，预计全年到位资金95亿元，同比增长11.6%。

产业结构不断优化。深入推进“两个转变”战略，“三农”工作亮点频现。粮食生产再获丰收，预计总产量63.1亿斤，同比增长15.4%。调增水稻、大豆、小麦等优质高效作物97.7万亩，种植北药7.1万亩，推广鸭稻、蟹稻、鱼稻9000亩。绿色食品种植基地面积550万亩，绿色有机食品认证面积315万亩。市级以上畜禽标准化示范场达105个，畜牧业实现产值66.9亿元，增长10%。推动农产品线上线下协同营销，建成中俄兴凯湖垂直购等7个电商平台，率先与省大米网完成对接，预计全年线上销售额1.2亿元。密山被列为国家农村产业融合发展示范园创建县，密山、虎林被列为全国水稻绿色高产高效创建示范县。深入推进“双轮驱动”战略，工业质量效益同步提升。1—11月份，规模以上工业企业实现主营业务收入191.7亿元，同比增长29.4%；利润15.4亿元，同比增加16.8亿元，均创五年来最快增速。全年净增规上企业22户。“两黑一绿一药”四大主导产业产值占规上工业比重的80%以上。石墨产业加速成长，企业发展到45家，石墨院士工作站2家，成立黑龙江乐新石墨烯研究院，建成省内首家等离子体生物质石墨材料重点实验室，国标委鳞片石墨分技术委员会即将落户鸡西。全省唯一石墨电商交易平台“墨都商城”上线运营，中国石墨产业创新发展高峰论坛在鸡西召开，石墨产业实现产业链、创新链、价值链、营销链一体发展，话语权和影响力大幅提升。深入推进“旅贸牵动”战略，第三产业繁荣活跃。牵头成立“龙江东部旅游产业联盟”，赴天津、上海、大连等城市开展营销推介，先后接待北京、广州、哈尔滨等省内外百人以上旅游团体50余个，客源市场辐射到长三角、珠三角、京津冀地区。引进首旅集团、泛华集团，对兴凯湖进行综合开发。兴凯湖—珍宝岛沿线被列入省重点推介的五条精品线路之一，珍宝岛景区被网民评为全省最受欢迎的十大景区之一。1—11月份，接待国内外游客991.6万人次，旅游收入64.4亿元，同比分别增长10.5%和17.1%。电子商务快速发展，自建电商平台16个，各类电子商务应用企业达236家，同比增长200%，实现网络交易额5亿元，同比增长160%。新引进金融企业3家，虎林双平米业等4户企业在双创板挂牌。1—11月份，金融机构各项存款余额1062.5亿元，贷款余额731.9亿元，存贷比68.9%，金融业服务地方发展能力不断增强。

改革取得重大进展。深化放管服改革，保留行政审批136项、行政权力3302项、行政责任3350项；梳理行政权力中介服务171项，保留规范105项；行政审批总时限由145个工作日压缩到109个。“多证合一、一照一码”商事制度改革取得新进展，企业实现“二十三证合一”，个体工商户实现“十三证合一”。深入推进事业单位分类改革，精简事业编制1556名，超额完成减控目标。完成了域内29家公立医院综合改革，各项医改指标排在全省前列。城乡居民基本医疗保险整合完毕，跨省异地就医实现直接结算。营改增试点、厂办大集体、国有林场等国家部署的改革任务顺利完成。国家确定的增量配电业务改革、鸡冠产城融合示范区建设、国有企业退休人员社会化管理等试点改革，扎实有序推进。大力度实施自我改革，在全省率先完成县域农信社产权制度改革，积极推进城市综合执法体制改革，完成市供水体制、供热补贴、市区间税收分享体制和经济开发区投融资体制等改革任务，发展活力不断释放。

民生改善成效显著。预计全年用于民生支出148.4亿元，占一般公共预算支出的79.8%。向人民承诺的10件实事完成节点任务。人民群众殷切期盼的一批好事顺利完成，采沉区棚户区改造工程15个新建项目全部建成，5.26万户居民喜迁新居；农村厕所革命走在全国前列，新增太阳能卫浴一体化5000户，累计达1.6万户，央视、新华社等各类媒体相继报道我市做法；更新公交车辆268台，矿区大供水一期、供热工程全部完成。脱贫攻坚扎实推进，当年精准脱贫643户，1586人。住房公积金政策延伸到自由职业者，实现全覆盖。人民群众反映强烈的一批难事得到有效化解，复工建设中心区城建滞留项目12个，回迁居民2337户；解决了6.3万户居民不动产权证不能办理问题；启动四海居社区、中山社区城市急危棚户区异地安置工作。人民群众高度关注的就业、教育、医疗、食品药品安全等一批大事取得新成效，城镇新就业4万余人，农民创业走在全省前列，密山、鸡东列入国家第三批支持农民工返乡创业试点。发放城乡低保金、特困人员供养金、城乡医疗救助金、残疾人“两项”补贴金、矿区困难职工救助等各类资金6亿多元，惠及居民近15万人。率先在全省整建制通过县域义务教育基本均衡国家验收，教育信息化应用在全省率先实现城乡学校全覆盖。完成家庭医生重点人群签约服务23.9万人，组建各类医联体11个，新建提档升级国家级标准化村卫生室30个。市中医院被省批准为国家中医住院医师规范化培训基地。食品药品安全监管连续四年被省政府评为“优秀”。鸡西版画馆建成使用，15幅版画入选全国工业版画研究院年展，3幅作品荣获创作奖。兴凯湖冰雪汽车拉力赛、中俄兴凯湖跨境马拉松赛等体育赛事影响力持续提升。全市涌现出亚冬会首冠满丹丹、全国公安楷模朱振龙、农民创业典型赵俊平，以及市供销社、市医院等一批国家级先进人物和先进集体。全市信访量、刑事案件发案量持续下降，社会大局保持稳定，人民群众的获得感、幸福感、安全感大幅提升。

城市建设全面提速。牡佳快客鸡西段开工建设，虎林支线机场推进顺利，国道鹤大公路滴道至麻山段大修完工，丹阿公路鸡穆界至鸡西改扩建列入国道升级改造“三年滚动计划”2018年项目库，城市对外交通更加顺畅。东升立交桥实现当年设计、当年建设、当年通车。改扩建中心区街路20条，新建停车场10处，新增停车位2000余个。完成燃气企业整合并购，中燃永久场站完工，英联燃气永久场站竣工供气；永昌东路等排水改造、兴国西路等园林绿化、东山街等路灯改造全部完成；新建改造垃圾中转站和地坑31座；打造城市管理重点示范街10条，城市的功能、品位、形象大幅提升。兴凯湖生态保护绩效考核连续两年优秀，获国家奖励资金8000余万元。全面落实“河长制”，全市设立四级河长1410人；在全省率先开展“水十条”考核评估，城市水源地水质达标率100%。集中整治大气污染，市区空气质量优良以上达标比例86.8%。植树造林4.3万亩，治理水土流失14.4万亩。桦木林场神洞山被批准为国家级森林公园，虎林获我省唯一的首批国家生态文明建设示范市称号，生态文明建设取得重要成就。

公共服务质效同升。坚决贯彻市委决策部署，主动接受人大、政协监督，定期向市人大汇报工作，向市政协通报工作，邀请人大代表、政协委员检查视察政府工作，认真办理人大代表、政协委员议案提案，已办理及列入规划逐步办理的人大代表议案、建议152件，办理率99%；政协委员提案146件，办理率98%。非涉密行政审批事项全部纳入网上办理，平均办理时间2.4天，提速率78.7%。建立市政府服务企业和项目建设“直通车”制度，为企业解决问题58件；市企业和创业投诉中心办理企业投诉案件35件；市政务服务中心投入运行，共受理各类业务3.9万件，办结率99.5%；完成公共资源平台整合，市公共资源交易中心挂牌成立，办理公共资源交易项目293个；开通“城市一号通”，累计受理群众诉求1.1万件，办结率96.9%。诚信鸡西建设取得新进展，在全国261个地级市城市信用状况监测中，我市排名上升到99位。

各位代表，过去一年取得的成绩来之不易。这是全市上下全面贯彻落实党中央国务院和省委省政府各项决策部署的结果，是市委统揽全局、科学决策的结果，是市人大、市政协有效监督、鼎力支持的结果，是全市上下团结拼搏、开拓进取的结果。在此，我代表市政府向全市广大干部群众，向所有关心、支持鸡西发展的同志们、朋友们，表示衷心的感谢，并致以崇高的敬意！

在看到成绩的同时，我们也清醒地认识到，工作中还有许多不足和挑战：产业规模不够大，层次不够高，转型发展的任务还很重；新经济、新产业、新业态等发展不足，新旧动能转换不快；企业创新力、竞争力、抗风险能力弱，新设立市场主体增速放缓；城市棚户区改造等民生保障还不充分，城市功能和管理水平与群众期盼还有差距；以煤矿为重点的安全生产监管工作压力大；政府系统工作人员的思想不够解放，专业素养、钻研精神、担当意识、工作效能有待进一步提高，等等。对这些问题，我们将高度重视，认真加以解决。

二、2018年工作安排和重点任务

2018年，是贯彻党的十九大精神的开局之年，是我市推进转型发展、决胜全面建成小康社会的关键之年。市委十三届二次全会描绘了新时代鸡西发展的蓝图。新时代、新目标要求政府要有新作为、新担当，我们将以一往无前的勇气，昂扬向上的斗志，务实求实的作风，全力推动鸡西转型发展再上新台阶。

今年政府工作的总体要求是：全面贯彻党的十九大精神，以习近平新时代中国特色社会主义思想为指导，按照省委十二届二次全会的战略部署和市委十三届二次全会确定的建设社会主义现代化新鸡西的目标，以稳中求进为总基调，以供给侧结构性改革为主线，坚定不移做强实体经济促转型，坚定不移实施三大变革提动能，坚定不移深化改革开放增活力，坚定不移提升群众福祉惠民生，坚定不移建设美丽鸡西优生态，加快转型发展步伐，努力为新时代的鸡西人民创造更加幸福美好生活。

综合考虑各方因素，全市经济社会发展的主要预期目标为：地区生产总值增长6.5%，规模以上工业增加值增长8%，固定资产投资增长6%，社会消费品零售总额增长8%，一般公共预算收入增长6%，外贸进出口总额增长10%，城乡居民人均可支配收入分别增长6.5%。

为做好政府工作，我们将更加注重贯彻新发展理念，以供给侧结构性改革为主线，推动质量变革、效率变革、动力变革，促进经济高质量发展；更加注重转型发展，全力实施“三大战略”，打造“一都五基地”，构建现代产业体系，在全省率先走出资源型城市转型发展新路子；更加注重新旧动能转换，推动产业项目、创新驱动与新增长领域培育相结合，做好“三篇大文章”，提升实体经济发展水平；更加注重规划引领，高起点编制城市发展规划，高水平建设管理城市，加强生态文明建设，树立文明城市形象，建设生态宜居文明美丽家园；更加注重人民群众的获得感，坚持以人民为中心，尽力而为、量力而行，办好民生实事，不断满足人民日益增长的美好生活需要；更加注重科学民主决策，依法高效行政，清朗廉洁从政，建设勤政廉洁务实高效政府；更加注重解放思想，深化改革开放，激发内生动力，勇于担当实干，狠抓工作落实，始终干在实处，走在前列。

重点抓好以下六个方面工作。

（一）贯彻新发展理念，筑强实体经济发展新引擎

实体经济是发展的根基。着眼高质量发展，坚持新旧动能同时发力，质量效率动力同步变革，推动实体经济提质增效。

打好项目建设攻坚战，走质量变革之路。产业项目是实体经济的重要载体。全年启动实施产业项目建设“五百工程”，即：投资5000万元以上项目100个，投资500万元—5000万元项目400个。重点推进唯大（鸡西）石墨烯产业园二期、鸡东30万吨燃料乙醇、密山翔晏玉米深加工等一批投资10亿元以上立市大项目；完成珍宝岛药业二期、超碳新材料超级电容器活性炭、三聚绿能十万吨炭基肥等一批投资5000万元以上的补链强链项目；开工建设中科遥感卫星产业应用、哈工大机器人集团碳基材料科创园、全丰航空植保无人机组装等一批科技型、成长型项目。进一步提高招商专业化水平，坚持大中小项目齐上，市县（市）区同抓，扎实开展产业链招商、专题招商、定向招商，签约项目、落地开工项目、到位资金实现新突破，引入更多新动能。加大项目推进力度，继续实行“3·15”“5·15”集中开工制、重点项目市级领导包保制、服务企业和项目建设“直通车”制、重点难点问题集中会办制、项目定期督办通报制。加快北汽普莱德石墨深加工及锂电负极材料等项目建设进度，全年28个项目实现竣工。

打好创新驱动攻坚战，走动力变革之路。创新是振兴实体经济的灵魂，是引领发展的第一动力。梳理四大主导产业技术需求，加强政府引导，激发企业主体作用，积极与清华大学、哈工大、航材院等科研院所合作对接，开展技术攻关，提升产业发展层次。推进清华大学鸡西石墨烯院士工作站、黑龙江省科学院鸡西石墨分院等院所建设，促进更多科技成果落地转化。实施新一轮科技型企业培育计划，新增主营业务收入500万元以上科技型企业10户，增长20%，新增科技型规上企业3户以上，至少再增加国家级高新技术企业1户。集合政府、黑龙江工业学院、黑龙江技师学院及企业力量，创办一批科技企业孵化器，鼓励、支持科技人员、大学生、农民、龙煤转移分流职工创业就业。新增省级以上科技孵化器、众创空间等创新平台4个。组织实施“市场主体培育三年行动计划”，推动新设立企业、个体工商户和专业合作社实现新发展，全年新设立市场主体增长10%以上。加强质量品牌建设，开展增品种、建标准、提品质、创品牌专项行动。全年高新技术产业产值增长9.3%。

打好要素保障攻坚战，走效率变革之路。大力实施园区提升工程，发挥好盛鑫投融资公司作用，强配套、补功能、夯基础、提水平，建设现代产业聚集发展平台。开工建设麻山石墨园区PPP项目，统筹推进梨树工业谷、鸡冠产业园、恒山石墨园区污水处理厂建设；完成鸡冠产业园、麻山石墨园区配套电源项目；加强鸡冠产业园区、麻山石墨园区、鸡东煤化工园区、密山经济开发区道路、排水等配套设施建设；完成梨树工业谷70万平方米、虎林经济开发区30万平方米土地收储。着眼于为实体经济提供全生命周期的金融服务，引导金融机构实施差别化信贷政策，加大对重点产业、重点企业以及成长型中小微企业信贷支持。引导企业利用好天使投资、风险投资、私募股权投资等多种金融工具。继续实施“企业上市直接融资培育计划”，发挥绿地鸡西企业挂牌孵化基地作用，鼓励企业对接资本市场，重点培育贝特瑞、普晨、浩市等企业挂牌上市。积极引导企业树立现代发展意识，加快建立现代企业制度。增强服务企业意识，积极构建“亲”“清”新型政商关系。大力弘扬企业家精神、劳模精神和工匠精神，造就一批引领鸡西发展的人才队伍。启动实施人才“十百千计划”，柔性引进10名行业领军人才，培育造就百名优秀企业家、千名鸡西大工匠，打造服务发展的人才高地。

（二）统筹三篇大文章，构建现代产业发展新体系

坚持质量第一，效益优先，以供给侧结构性改革为主线，做好“老字号”、“原字号”、“新字号”三篇大文章，构建支撑转型发展的现代产业体系。

“双轮驱动”四大主导产业。升级发展石墨产业。加快唯大、贝特瑞等企业石墨续建项目建设，尽快释放产能。启动建设华东石墨深加工及煤层气制碳纳米管、浩市圣莎拉高品级钻石材料等项目，推动石墨产业向中高端迈进，石墨加工转化率达到60%以上。合理增设采矿权，完成6个历史遗留矿权“一体化”招标。加强石墨产业科技创新，建设石墨“技术银行”，石墨制品检验检测能力实现全覆盖，国际标准参数达到50%以上，增强产业发展话语权。坚持以品牌促营销，制定区域性石墨产品质量标准体系，推进同一产品同一标准，叫响“鸡西石墨”区域品牌。加强“墨都商城”电商平台建设，拓展营销渠道，全年网络交易额达到10亿元以上，打造石墨产品交易中心。延伸发展煤炭产业。围绕“煤头电尾”、“煤头化尾”，开工建设大唐热电8.5万千瓦背压机组，支持启动天和焦化、天诚镁业开工生产，持续跟进中小型现代精细煤化工项目；完成地方煤矿兼并重组，推进原煤能洗尽洗，提高煤炭产品附加值。精深发展食品和农副产品加工业。围绕“粮头食尾”，发展水稻、大豆、红小豆、紫苏、食用菌、蜂产品、畜产品等深加工，推进农副产品加工多元发展、规模发展、深度发展；围绕“农头工尾”，推进鸡东、密山玉米燃料乙醇、玉米酒精、秸秆综合利用等项目，延长产业链，提升价值链。规模发展生物医药产业。坚持集群化发展、规模化种植、品牌化营销，完成珍宝岛药业二期建设，启动三期工程；推动新医圣蒲参盆腔泰项目尽快投产达效。谋划推进鸡东、梨树北药加工项目，带动北药种植8万亩以上。加强药材流通体系建设，打造北药销售联合体，推进珍宝岛药业梨树山产品大市场提档升级。围绕“四大主导产业”，促进企业裂变升级，全年净增规上企业10户。支持鸡西煤机厂恢复生产，盘活碳素厂、密山啤酒厂等闲置企业资产。

“旅贸牵动”现代服务业。大力发展旅游业。坚持“一核”抓合作、“三区”抓建设、品牌抓营销，推动旅游业加快发展。以兴凯湖为核心，围绕打造中俄环兴凯湖生态旅游康养度假带，加快完善规划体系，深化与泛华集团、首旅集团合作，综合开发兴凯湖，同步带动乌苏里江、虎头、珍宝岛旅游。围绕北大荒文化旅游区、完达山生态旅游区、城市休闲旅游区三大板块，加快兴凯湖水上休闲游乐项目和各景区开发建设步伐。推动兴凯湖景区晋升4A级景区，创建虎头珍宝岛5A级景区。坚持以市场需求倒逼旅游产品供给，全方位、多角度深化营销。重点加强与俄罗斯远东地区的联动营销、龙江东部旅游产业联盟城市的集成营销，进一步叫响“不一样的江湖”旅游品牌。深入推进旅游与文化、体育、健康养老等产业融合发展，办好兴凯湖冰雪汽车拉力赛、中俄跨境马拉松等精品赛事，集中打造“兴凯湖旅游”名片。加强旅游配套设施建设，完善吃住行游购娱要素，提高服务能力和接待水平，树立鸡西旅游新形象。加快发展现代物流业，重点推进泛华物流园区、烟草物流园区、滴道一体化智慧物流综合产业园区、密山20万吨冷链物流及生猪屠宰等项目建设，建成一批生产服务型物流园区和配送中心。促进快递业进一步发展。积极发展健康产业，谋划推进社会福利院失能护理楼、市养老康复指导中心建设。引进社会资本，推进鸡冠区养老指导服务中心启动运营。促进房地产业、金融业健康发展。侯卫东官场笔记

多元促动新经济新产业。实施电子商务提升工程。全面落实电商发展政策，支持引导电商经营主体加快发展。引进专业营销队伍，提升本地平台营销能力，全年网上销售额实现12亿元以上。开工建设密山跨境电子商务综合园区、滴道跨境电商产业园。实施新产业倍增工程。继续组织实施“新增长领域三年滚动发展计划”，不断挖掘、培育、释放优势和潜力，动态推动30个新增长领域发展，生成一批新项目，培育新动能。依托中科遥感卫星产业应用项目，加快推进大数据、云计算、物联网技术向各行业融合渗透。精准把握分众化需求，培育发展分享经济、楼宇经济和数字经济。

（三）实施乡村振兴战略，锚定农业农村发展新方位

以打造农业强市为目标，深入实施“两个转变”战略，促进农村一二三产融合发展，实现农业强、农民富、农村美。

持续调优产业结构。坚持稳粮、优经、扩饲，经济作物和饲料作物种植面积达到20万亩以上。深入实施“三减”行动，增加中高端农产品供给，推广鸭稻、蟹稻、鱼稻1.5万亩。绿色食品种植基地面积达到575万亩。着力推进“两牛一猪一禽”规模化标准化养殖，新建市级以上标准化示范场10个。坚持多元化市场供给，发展近郊农业，蔬菜、瓜果、鲜食玉米等种植面积达到10万亩以上；积极发展林果、林蜂、食用菌等林下产业，林果面积达到2.1万亩，林蜂6.5万箱；以白鱼、河蟹为重点的水产养殖面积达到35.5万亩。

加快产业融合发展。深入推进农工融合，落实“两头两尾”方案，围绕高品质谷物食品、乳制品、畜产品以及特色饮品，发展农副产品精深加工，龙头企业达到110户。深入推进农旅融合，发展观光农业、体验农业，打造白鱼湾、虎头镇、城子河丰安特色民俗村寨等一批特色村镇。深入推进农商融合，借助淘宝网、兴凯湖垂直购等域内外电商平台，鼓励合作社、家庭农场、创业农民开办特色网店，壮大农村电商群体。对接市场需求，用好营销“16条路径”和“四种模式”，加强农产品营销，使农民在价值链上获得更多收入。积极培育多元融合主体，农民专业合作社、家庭农场、农业企业和产业联盟等新型农业经营主体发展到7910户，参与创业农民达到7万人。探索建立以龙头企业为引领、家庭农场为基础、农民合作社为纽带的农业产业化联合体，从生产、社会化服务到加工流通形成完整产业链条。支持滴道绿色蔬菜园创建省级农业科技示范园区。

强化生产要素保障。推广水稻节水增效、测土配方施肥等农业适用技术，科技贡献率达67.5%。大力发展设施农业，建设“互联网+”高标准示范基地18.5万亩，农业综合机械化率达97%。落实政府主体责任，建立完善粮食安全责任体系。深入推进农业改革，完成农村土地确权登记，稳步推进土地流转，规模经营面积达到345万亩；完成农村集体产权制度改革试点工作，大力实施“金鸡归巢”计划，吸引企业家投资乡村产业项目，壮大村集体经济，释放农业农村发展活力。

实施精准脱贫攻坚。突出产业扶贫，围绕“一村一品”，每个贫困村至少建成一个脱贫产业项目。围绕教育、医疗、住房“三保障”目标，落实好国家和省系列助学政策，确保贫困学生不因贫困而失学；建档立卡贫困人口实现家庭医生签约服务和9种大病集中救治全覆盖，实施商业医疗补充保险，减轻贫困户医疗负担；改造农村危房463户。全年精准脱贫890户、2006人。

建设美丽宜居村庄。开展农村人居环境整治三年行动，以农村太阳能卫浴一体化建设为载体，继续推进农村厕所革命。加强农村垃圾、污水集中收集、无害化处理，从源头控制对乡村的污染。加快农村饮水安全工程建设，解决137个村屯7.8万农民饮水安全问题。加强农村公路硬化、村屯绿化美化净化，倡导乡风文明，让农村成为幸福文明宜居的美好家园。

（四）深化改革开放，释放城市转型发展新活力

高举改革开放旗帜，坚持以改革破解发展难题，以开放释放发展活力。

深化重点领域改革。坚持以改革提效能，深化“放管服”改革，依托“互联网＋政务服务”，持续推进行政审批流程再造。全面实施市场准入负面清单制度。深化商事制度改革，推进企业登记全程电子化，为创业者提供便利服务。实施安全生产领域改革，提高安全生产整体水平。盘活政府系统非办公类资产，将更多的公共资源配置到生产领域。实施重要资源市场化配置。坚持以改革降成本，稳步推进国企“三供一业”职能分离移交、国企退休人员社会化管理试点等改革。深入推进矿区增量配电业务改革，售电公司正式运行。坚持以改革活机制，深化医药卫生体制改革，扎实推进城市执法、公交、供水体制改革，完成盐业体制改革。落实住房提租补贴政策。稳步推进事业单位分类改革。进一步理顺经济开发区管理体制机制。创新人才培养引进使用激励保障机制。

全面扩大对外开放。加大工作力度，推动口岸经济实现突破性进展。进一步优化虎林口岸通关环境，高质量运行虎林中俄互市贸易区，放大口岸竞争优势；加快推进密山口岸液化石油气进口、大豆回运加工等项目建设，确保两口岸过货超5万吨、过客超5万人次的“双超”目标。加强跨境产业园建设，落实口岸奖励政策，培育壮大远华、松海等外经贸主体。加强与俄远东地区的交流合作，举办好中俄共度新年、中俄文化艺术交流、“旅游+”等活动。紧紧抓住与广东肇庆市对口合作的机遇，重点在产业发展、园区建设、人才培养等方面主动对接，深入交流，向先进学习，提升发展能力。

推进区域协调发展。引导各县（市）区立足自身区位条件、资源禀赋，刻画特色鲜明的主导产业，形成错位发展、优势互补的发展格局。鼓励三县（市）晋位发展，瞄准全省十强目标，力争在全省县域综合排名中晋位升级。支持各区争先发展，落实市区间税收收入分享制度，推动六区大上立区项目、财源项目，打造工业强区、财税富区。继续推进鸡冠产城融合示范区建设，发挥示范引领作用。加强与农垦、森工、龙煤及电力、通讯、金融等中省直单位的合作，推进携手发展。

（五）实施五大工程，建设幸福美丽宜居新鸡西

以功能优、城市美、管理强、生态好为方向，推动城市建设与管理上水平，城市面貌实现大变样。

实施城际交通提速工程。加快牡佳快客鸡西段建设，开工建设虎林支线机场，实施兴凯湖机场改扩建工程，推进鸡东德胜路、恒山三矿区、梨树四道街等5处跨铁立交桥建设，争取城子河环线纳入国家“十三五”规划，努力构建内联外通、便捷高效的城市交通体系。

实施中心区提质工程。完善城市功能，铺设燃气管线20公里，管网覆盖面达10万户；改造市区老旧供热管网12公里；实施第三净水厂及供水管线改造项目；建设腾飞桥、园林小区等5项排水工程；改造垃圾中转站3座。改善出行环境，改造东升花园、龙域嘉苑等6个小区周边道路，建设停车场6处、停车位2000个。加强会展中心、版画基地等10处地域美化绿化，完成广益街、佳和路等4条街路亮化，进一步提升城市形象品位。全力化解城建历史遗留问题，中心区剩余4447户回迁安置问题基本解决，不动产办证问题基本解决。

实施中心镇提档工程。做好采沉区棚改收尾工作，推进虎林、鸡东等城市棚户区改造。新建麻山站前路、民主路，改造密山长春街、虎林建设街等街路，建成滴道南外环路，完成鸡东大桥、恒山民艳桥等桥梁改造。新建改造鸡东、密山、虎林、梨树供水、污水处理工程，开工建设麻山引水工程，完成矿区大供热大供水项目。开工建设虎林穆棱河水上公园、恒山文化体育活动中心。

实施城市管理提升工程。坚持规划引领，做好第四轮城市《总体规划》修编工作。以鸡冠产城融合示范区建设为契机，探索推进中心区“多规合一”。高水平规划管理利用采沉区腾空土地。加强城市综合化管理，发挥城市管理工作联席会议协调作用，建立健全多部门联动机制，强化综合执法，部门协调配合，反应迅速及时，确保城市管理到位。加强城市精细化管理，综合整治中心大街等15条重点街路，着力解决商业区附近、医院学校周边交通拥堵问题。专项治理小区楼宇乱贴乱画、环境卫生脏乱差等影响市容市貌问题。加强小区业主委员会建设，多方筹措资金，加强老旧小区改造，提升物业管理水平。推进全国文明城市（提名）创建工作，大力倡导文明执法、文明服务、文明出行、文明驾驶、文明经营、文明祭祀等行为，形成树文明新风、做文明市民氛围，提升城市文明形象。

实施生态文明提效工程。开展“治霾卫蓝”行动，严格落实“大气十条”，加强重点领域集中整治，推进煤炭清洁高效利用和散煤治理，加强秸秆限烧禁烧及综合利用。开展“治污净水”行动，落实河长、湖长制，完成87条重点河流一河一策编制工作。严格落实“水十条”，以穆棱河为重点，专项整治河湖“八乱”。开展“保土护田”行动，严格落实“土十条”。加强兴凯湖等6个自然保护区的综合管理，理顺体制机制，严厉打击乱侵乱占、毁林开荒、非法捕捞等行为。推动珍宝岛湿地自然保护区加入“世界人与生物圈保护区网络”。全年植树造林1.95万亩。

（六）坚持以人民为中心，满足群众美好生活新需要

从人民群众最关心最直接最现实的问题入手，补短板，强弱项，既尽力而为，又量力而行，多办实事好事，多解难事忧事，让我们的城市更温暖，让人民群众更幸福。

倾心办好重点8件民生实事。（1）改造城市棚户区13170户。（2）改造全市老旧小区12个，楼房89栋；推进“三供一业”二期工程建设，改造矿区住宅楼360栋。（3）推进“厕所革命”，改造建设城市水冲公共厕所65座，新增农村太阳能卫浴一体化用户6000户。（4）开工建设国道丹阿公路鸡穆界至鸡西段及延长线和高铁连接线。（5）实施永昌路、鸡密南路、电工路等10条中心区街路改造工程。（6）开工建设鸡西室内滑冰馆。（7）建成市职业技能公共实训基地，打造实用型人才培训和创业孵化平台。（8）鸡西静脉产业园竣工投产，提高城市生活垃圾无害化、减量化、资源化处理水平。

全面发展社会事业。实施创业就业工程，完善创业就业培训服务体系，提高全社会就业水平，实现城镇新就业3.8万人。实施教育优先工程，建成鸡西腾飞学校、城子河树英小学，改扩建鸡东二中，启动黑龙江工业学院新图书馆、大学生创新创业园和黑龙江技师学院现代服务专业实训楼建设。规范幼儿园办园行为，提高中小学教学质量，支持黑龙江工业学院建设一流应用技术大学，提升职业教育服务产业发展能力。实施新高考改革。加强师德师风建设，提升教师队伍专业素质。全年资助贫困学生3万人次。实施全民健康工程，深化公立医院改革，加快市医院“3+2+1”医联体建设，重点人群家庭医生签约覆盖率提高5个百分点以上。完成市医院新门诊、医技和综合楼项目建设。实施文化惠民工程，积极发展文化事业，创作一批反映鸡西历史文化题材的文艺精品。推进版画产业化发展，打造集版画创作、展示、营销为一体的特色文化产业创研中心，叫响“中国版画艺术之乡”文化品牌。推进文化市场综合执法改革。实施体育强基工程，加强三县（市）综合体育场馆建设，打造城市15分钟健身活动圈，实现行政村体育健身场地设施全覆盖。办好城市公路自行车赛、中俄跨境马拉松等赛事活动，组织参加黑龙江省第十四届运动会。实施社区创建工程，新建城乡标准化社区12个，完善社区服务功能，提升服务水平。推动军民融合发展，开展拥军优属、拥政爱民“双十”活动，争创全国双拥模范城“八连冠”。

全力维护安全稳定。突出抓好煤矿安全生产，严格落实省委煤矿整治整合与化解过剩产能专项巡视督查反馈意见，切实整改兼并重组进展缓慢、安全生产责任落实不到位等问题。严格落实企业主体责任、政府属地责任和行业监管责任，强化煤矿安全专项整治，打通安全生产最后一公里。加强非煤矿山、危险化学品、烟花爆竹、建筑施工、森防消防、特种设备、公共场所等重点行业、领域安全监管，严厉治理超载超限和酒驾毒驾，坚决防范遏制各类重特大事故发生。加强食品药品安全监管，确保人民群众舌尖上的饮食用药安全。深化“平安鸡西”建设，做好信访积案和矛盾化解工作，加强社会治安综合治理，严厉打击各类违法犯罪活动，确保社会安定稳定。

三、不忘初心，牢记使命，建设人民满意的服务型政府

新时代赋予新使命，新征程期待新作为。我们将始终同全市人民想在一起、干在一起，持之以恒转作风、提效能、强本领，努力打造“干实事、真干事、干成事”的人民满意政府。

始终坚持正确的政治方向，提高站位、忠诚履职。深入贯彻落实党的十九大精神，以习近平新时代中国特色社会主义思想武装头脑，开展“不忘初心，牢记使命”主题教育，牢固树立“四个意识”。坚决维护以习近平同志为核心的党中央权威和集中统一领导，贯彻落实国务院、省委、省政府和市委决策部署，严守党的政治纪律和政治规矩，提高政治站位。贯彻落实新发展理念，践行以人民为中心的发展思想，创造党和人民满意的工作业绩。

始终坚持昂扬的奋斗姿态，解放思想、担当实干。敢于破除一切落后的思想禁锢，树立与新时代相适应的发展理念和思想观念，提升干事创业的精气神，激情工作，担当作为，勇创佳绩。立足“政治过硬、本领高强”，持续深化政府专业知识学习，切实提升8大本领，打造“学习型政府、专家型队伍”。充分发扬“钉钉子”精神，建立“马上办、坚决办、一次办、公开办，想干、会干、敢干、实干”抓落实的工作机制，工作任务做到细化量化具体化，责任到人，细化到事，跟踪督办，问责不办，打造上下贯通、层层落实的责任链条，提高执行力，确保各项部署落地生根。

始终坚持法治的尊严权威，真诚服务、依法行政。坚持把遵守宪法和法律作为施政根本，深入推进“七五”普法，全面提高政府系统工作人员的法治思维和依法行政能力，严格按照法定权限和程序用权履责，全力推进法治政府建设。深化政务公开，自觉接受人大、政协和社会各届监督，切实提高行政公信力，打造阳光政府。推动诚信体系建设，以政务诚信带动商务诚信和社会诚信，形成良好的社会风尚。坚持以企业和群众的感受为标尺，少讲不行的理由，多想能干的办法，不断优化网上和政务大厅服务，让流程跑、数据跑、政府跑，跑出便民便企的加速度，实现服务发展的新高度。

始终严守纪律的刚性约束，勤勉为民、廉洁从政。以永远在路上的坚韧，锲而不舍抓作风建设，全面落实党风廉政建设主体责任和“一岗双责”，持之以恒正风肃纪，坚决反对“四风”。坚持以上率下，严格遵守“八项规定”等党内各项法规，始终保持廉洁本色。树立过紧日子思想，强化财政预算管理，推行公共资源项目电子化招投标等措施，提高政府资金使用效率。加大公共资金、国有资产、国有资源监管力度，强化重点领域和关键岗位监督，严厉整肃庸政、懒政、怠政行为，打造勤政清廉政府。

各位代表，使命呼唤担当，奋斗铸就辉煌！让我们紧密团结在以习近平同志为核心的党中央周围，在省委、省政府和市委的坚强领导下，不忘初心、牢记使命，踏实苦干、奋勇争先，为建设富强民主文明和谐美丽的社会主义现代化新鸡西而努力奋斗!
